# Estimating the Health Effect of Cigarette Smoking Duration in South Africa

**DOI:** 10.3390/ijerph192013005

**Published:** 2022-10-11

**Authors:** Alfred Kechia Mukong

**Affiliations:** Department of Economics, University of Namibia, Private Bag 13301, Windhoek 13301, Namibia; amukong@unam.na

**Keywords:** cigarette smoking, smoking-related diseases, regression discontinuity design, South Africa

## Abstract

This paper deepens the empirical analysis of the health effects of smoking by using the average treatment effect on the treated (ATET) and regression discontinuity design (RDD) to estimate the impact of smoking duration on health. The paper estimates the effect of cigarette smoking on health, that is, the exogenous increase in the probability of smoking-related ill health when individuals smoke up to a certain number of years. Using the National Income Dynamic survey (NIDS), the study finds that the probability of reporting poor health and/or suffering smoking-related diseases increases with the years of smoking. The magnitude of the effect is higher when smoking-related diseases rather than self-assessed health is considered but varies across time, socioeconomic status, and with different health outcomes. The effects are robust under several different parametric and non-parametric models. Using RDD, the paper also finds evidence of a discrete jump in poor health when individuals smoke up to 30 years. The results suggest that policies that are designed to reduce current levels of cigarette smoking may have a desirable impact and can create both current and future public health benefits.

## 1. Introduction

Since the United States Surgeon General’s Report linked cigarette smoking to lung cancer and cardiovascular disease in 1964, the list of smoking-related diseases has grown, and cigarette smoking is recognized as one of the leading preventable cause of death in the world. Smoking harms virtually every organ in the body, contributing to an increase in the burden of non-communicable diseases worldwide [1,2]. Smoking is responsible for cancer in multiple organs, cardiovascular diseases, respiratory diseases, reproductive effects, and many more harmful health effects [3,4,5,6]. Smoking accounts for 6.3% of the total burden of diseases worldwide [7] and imposes an enormous economic burden on world economies through its harmful effects on health, cost of medical care, and productivity loss [8,9,10,11]. Specifically, cigarette smoking costs world economies over USD 1 trillion annually in health care expenditure and productivity losses [12] and kills over 7 million people yearly [13]. In South Africa, smoking-related diseases remain one of the leading causes of mortality [14]. However, the health effects of smoking are not immediate, and evaluating the role of smoking duration on related health outcomes is vital for effective policy options.

Studies have shown that smoking duration (years of smoking) rather than smoking intensity (average number of cigarettes smoked per day) is a good predictor of smoking-related morbidity and mortality. For instance, [15] showed that a three-fold increase in smoking intensity may produce a three-fold rise in the risk of lung cancer, but a three-fold increase in smoking duration might produce a 100-fold increase in the risk of lung cancer. In addition, individuals with smoking onset at age 15 and smoking one pack a day for 40 years have higher risk of lung cancer than those with onset at age 35 and smoking two packs a day for 20 years [15]. On average, the health damage from smoking usually emerges at least 20 to 30 years after exposure [16]. The long delay between the age at smoking onset and related morbidity and mortality suggest that current levels of morbidity and mortality largely reflect past smoking patterns, while future levels depend on current and future smoking patterns. Thus, the role of smoking duration might be important when assessing the health effects of tobacco use. There is evidence of the relationship between smoking duration and related diseases, but focus has been on some specific diseases [6,17,18,19]. However, there is a scarcity of studies on the health effects of smoking duration in developing countries, and knowledge from reviewed studies suggest that no study has analysed this relationship in South Africa. This study addresses this critical evidence gap by examining the hazardous effects of smoking duration on related health outcomes in South Africa. The paper considers comprehensively a number of smoking-related diseases and individual characteristics that are important in explaining variation in health status.

The health effects of smoking in South Africa are extensive and well-documented [20,21,22]. Although the prevalence of smoking is declining [23], the relative risk of death for current and former smokers continues to rise [10]. Smoking caused 36% of all adult deaths in 2015 and accounts for over 170,000 deaths annually, a tremendous increase from 44,000 [24,25]. Some studies, although few, have demonstrated that smoking increases the probability of related morbidity in South Africa [22,26]. However, this is one of the first studies to empirically estimate the health effects of smoking duration in South Africa. The study compares the health outcomes of individuals below and above a given range of years of smoking, isolating the effect of smoking duration on health. A comprehensive review of smoking-related diseases provides a better picture on the effect of smoking duration on health.

## 2. Material and Methods

### 2.1. Data and Key Variables

This paper draws on data from the South African National Income Dynamic Study (NIDS), which are publicly available data that provide information on smoking patterns and smoking-related diseases. NIDS is a nationwide representative panel data conducted after every two years. The analysis is based on the first four waves collected between 2008 and 2015. Information on smoking duration (years of smoking) is not widely available in many datasets, including the one used in this paper. The paper, therefore, used the difference between the age of the individual at the time of the survey and the age at which they started smoking to measure smoking duration. Lifetime smoking information of individuals was categorized in much detail as sample sizes allowed: smoking duration (years, ≤10, 10–19, 20–29, and 30+). Non-smokers are used as the base category. Based on existing literature and data availability, smoking-related health indicators used in the analysis include tuberculosis, stroke, cancer, heart problems, high blood pressure, persistent cough, and chest pain. Nicotinic receptors are found throughout the body, such as in the brain, muscle, lungs, kidneys, and skin [27], and are associated with the highlighted health outcomes. Smoking is a strong predictor of tuberculosis [28], lung cancer [29], cardiovascular heart diseases [30], incidence of stroke [31], and depression [32]. The health outcomes are based on medical diagnoses and are coded as 1 if diagnosed with a particular disease and 0 otherwise. For a more generic measure of smoking-related health outcomes, a composite health index is constructed using the min–max rescaling transformation procedure. This procedure helps to reduce the dimensions of a data set where there are large numbers of observed variables that are thought to reflect a smaller number of underlying or latent variables. This method has been used widely is constructing indices [33,34,35]. The aim was not to interpret the index but to generate or recover a variable that can be used in the regression. Self-assessed health is one of the closest measures that captures all dimensions of health and is a strong predictor of mortality [36,37]. A sensitivity analysis is conducted by investigating the effect of smoking duration on self-assessed health. Self-assessed health takes the value of 1 if an individual reported poor health and 0 if excellent health.

### 2.2. Empirical Strategy

First, the paper estimates the effect of smoking duration on the composite index of smoking-related health outcomes using an ordinary least square (OLS) and on self-reported health using a probit model (Equation (1)). The assumption is that smoking above a certain period increases the probability of ill health. Existing evidence suggests that this probability is significantly high for individuals who have been smoking for at least 20 to 30 years [16]. However, this paper estimates the effects of smoking duration by comparing the health outcomes of individuals below and above the different smoking duration cut-offs. The model is specified as follows:

(1) $$h_{i}=\beta_{0}+\beta_{1}S_{i}+\beta^{\prime }X_{i}+\varepsilon_{i}\;$$

where $h_{i}$ measures health status, $S_{i}$ smoking duration, and $X_{i}$ is a vector of observable individual characteristics such as household income, religion, educational attainment, marital status, gender, age, race, and employment status. These controls are based on variables that are known, empirically or theoretically, to be associated with health. The inclusion of these variables helps increase the precision of the estimates. The estimates of smoking duration themselves do not tell us anything about the size of the effects of smoking on health. To recover the impact of smoking on health outcomes, the study estimates the average treatment effect on the treated (ATET), specified as follows:

(2) $$\tau_{ATET}=E\left[Y_{1i}-Y_{0i}/D_{i}=1\right]=E\left[Y_{1i}/D_{i}=1\right]-E\left[Y_{0i}/D_{i}=1\right]$$

where $E\left[Y_{1i}/D_{i}=1\right]$ is the average health status of smokers, and $E\left[Y_{0i}/D_{i}=1\right]$ is the health status of smokers had they not been smokers, which is not observable. The study used a control group and regression adjustment inverse-probability weighting to estimate consistent estimates of ATET.

A regression discontinuity design (RDD) was used to model the non-parametric function of smoking duration. Following Yörük and Yörük [38], local linear regressions were used for the non-parametric specification to estimate the left and right limits of discontinuity at any given years of smoking. According to Yörük and Yörük [38], the general model with different degrees of polynomials that are fully interacting with the treatment variable (years of smoking) is given by:

(3) $$H_{i}=\beta^{\prime }X_{i}+\delta T_{i}+\overset{k}{\underset{j=1}{\sum }}\alpha_{j}years_{i}^{j}+\overset{k}{\underset{j=1}{\sum }}\omega_{j}\left(T_{i}*years_{i}^{j}\right)+\varepsilon_{i}\;$$

where *k* = {1,2}, and *δ* is the coefficient of interest, indicating the effect of smoking duration on the relevant health outcomes. This can be interpreted as the causal effect of a marginal increase in smoking duration on health of individuals who smoked up to the number of years at the cut-offs. The difference between the two limits is interpreted as the local treatment effect of smoking duration at the cut-off on health. The triangular kernel is used because it puts more weight on observations closer to the cut-off point and is boundary optimal [39]. There is no generally agreed method for the selection of optimal bandwidths in the non-parametric RDD. The optimal bandwidth selection procedure suggested by Imbens and Kalyanaraman [40] was observed to be extremely small and under-smooths the data [38]. A broad range of candidate bandwidth suggested by Yörük and Yörük [38] was used. The data were analysed using the Stata Version 15 software (Quantec, Pretoria, South Africa).

## 3. Results

### 3.1. Descriptive Statistics

Table 1 presents descriptive statistics for both smoking behaviour and smoking-related disease profile of the studied population. The descriptive statistics focused on the latest wave (Wave 4) of the data used. The results indicate that smoking participation rate as of 2014 in South Africa was 20%. The average years of smoking per smoker was 17.34 years. The results further suggest that 4%, 11%, and 4% of the study’s population and 6%, 10%, and 3% of the smoking population are, respectively, suffering from tuberculosis, high blood pressure, and diabetes. In addition, 1%, 2%, and 13% of the study’s population are diagnosed of stroke, heart disease, and persistent cough relative to 1%, 2%, and 17%, respectively, for smokers. Depression has the highest mean values, ranging from 45% for the study’s population and 51% for the smoking population. The proportion of self-reported poor health is 10% and 13%, respectively, for the entire population and smoking sample, whereas the health index for smoking-related diseases suggests a deterioration when individuals are smokers.

### 3.2. Empirical Results

Table 2 shows the effect of smoking duration on health outcomes after controlling for individual characteristics. Compared with non-smokers, smokers are more likely to report poor health and/or suffer from smoking-related diseases. Based on Wave 4, and relative to non-smokers, the probability of reporting poor health is 3.7%, 3.6%, and 4% for individuals who have smoked for 10 to 19 years, 20 to 29 years, and over 30 years, respectively. The results are insignificant for those who have smoked for between 0 and 10 years. The index score of suffering from smoking-related health outcomes significantly increases by 0.13, 0.15, 0.12, and 0.31 units for those who have smoked for less than 10, 10 to 19, 20 to 29, and over 30 years, respectively (Wave 4). The effects are consistent from Wave 1 to Wave 3. The magnitude of the effect is generally higher for those who have smoked for at least 20 years, suggesting that the negative effect of cigarette smoking on health increases with the years of smoking.

Based on the ATET estimates in Table 3, smokers are significantly more likely to report poor health and/or be diagnosed of smoking-related diseases compared to non-smokers. The estimates for smoking-related health index are higher than those from self-assessed health. In Wave 4, the estimated ATET for smoking-related disease is 0.103 and 0.024 for self-assessed health. This suggests that the probability of reporting poor health and the index for the prevalence of smoking-related diseases are 2.4% and 0.103 units higher among smokers than non-smokers. The results are consistent across Wave 1 to Wave 3, where the estimated effects are higher when smoking-related diseases rather than self-assessed health are considered. Estimates of ATET by gender focused only on smoking-related diseases. The magnitude of the effect by gender is inconsistent across the different waves. The male estimates are slightly greater in magnitude when Wave 1 and Wave 2 are considered, while the female estimates are greater when Wave 3 and Wave 4 are considered.

In Table 4, the interest was in estimating the impact of smoking on related diseases by race and level of education. The results focused only on the index of smoking-related diseases. In Wave 4, the estimated effects of smoking on related diseases are significant among the Black population and the coloured population but insignificant among the White population. However, in Wave 1, the effect of smoking is significant across all population groups. The results are insignificant for the coloured population in Wave 2 to Wave 3. The magnitude of the effect by education are inconsistent across the different waves. The estimates for those with at most primary education are slightly greater in magnitude in Wave 1 and Wave 2, while the estimates of those with tertiary education are greater in Wave 3 and Wave 4. This suggests that the health benefits for reducing smoking varies significantly with the socioeconomic status in South Africa.

**Figure 1:**
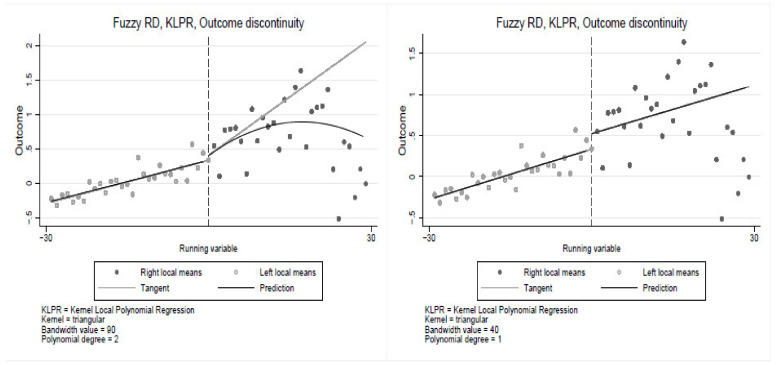
Regression discontinuity estimates for the entire sample.

**Figure 2:**
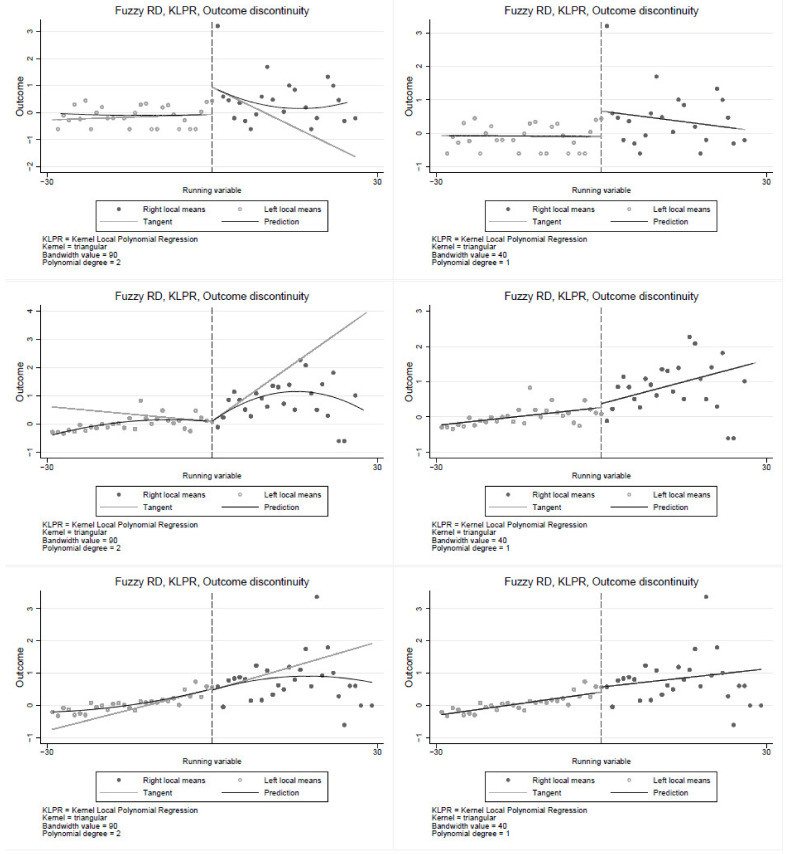
Regression discontinuity by race.

**Figure 3:**
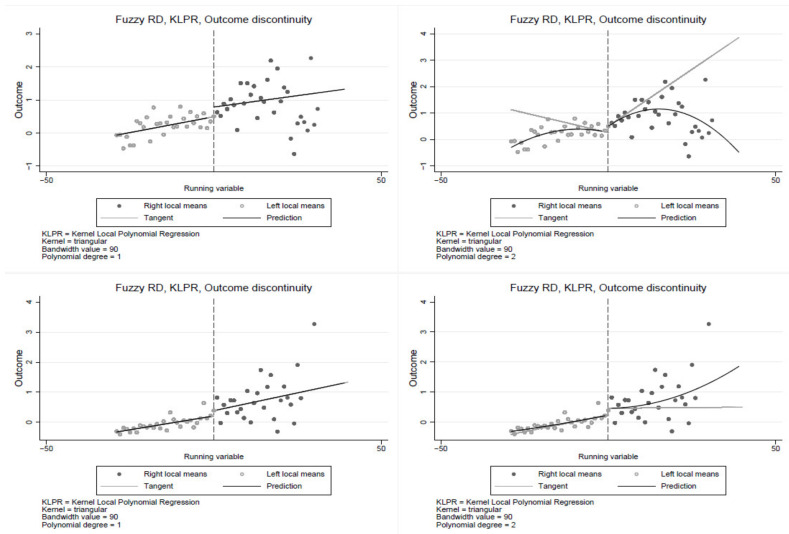
Regression discontinuity by education.

**Figure 4:**
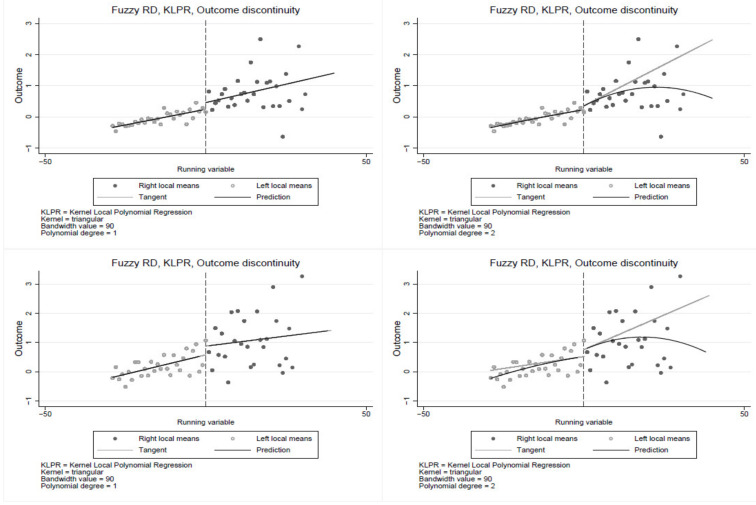
Regression discontinuity by gender.

The RDD non-parametric specification is estimated using triangular kernel with a bandwidth of 90 and cut-off at 30 years of smoking. The results in Figure 1 show a significant discontinuity at the cut-off, suggesting that smoking duration is a significant determinant of poor health, confirming estimates from the parametric models. The non-parametric estimates yield similar results, indicating some percentage point jump in poor health at the cut-off. The results in Figure 1 clearly show the jump in poor health at the 30 years of smoking cut-off. Consistent with the parametric results in Table 4, the first row of Figure 2 shows a relatively large jump in poor health at the cut-off among the Black population compared to the coloured and the White populations. Similar results are observed in Figure 3 and Figure 4, where the jump is relatively larger among those with primary education and the male sub-group, respectively. The results show that smoking duration affects the health of both males and females. However, the effects are significantly higher among male than female smokers who have smoked for at least 30 years. Thus, the results are robust under both the parametric and non-parametric models. These findings are particularly important given the ongoing public policy debates about a stricter tobacco control target for nations.

This figure is an estimate from the entire population of smoking individuals. In the first column, a quadratic polynomial is used, whereas in the second column, a linear polynomial is used. The cuff-off point is 30 years of smoking (to the right of each graph are individuals who have smoked for at least 30 years, and to the left are smokers with less than 30 years of smoking). The jump at the cut-off point shows the impact of smoking on health between the two groups.

The first row is for the White/Indian, second for the coloured, and the third is for the Black population. In the first column, a quadratic polynomial is used, whereas in the second column, a linear polynomial is used. The cuff-off point is 30 years of smoking (to the right of each graph are individuals who have smoked for at least 30 years, and to the left are smokers with less than 30 years of smoking).

The first row is for individuals with less than secondary education, and the second is for individuals with at least secondary education. In the first column, a linear polynomial is used, whereas in the second column, a quadratic polynomial is used. The cuff-off point is 30 years of smoking (to the right of each graph are individuals who have smoked for at least 30 years, and to the left are smokers with less than 30 years of smoking).

The first row is for male, and the second is for female. In the first column, a linear polynomial is used, whereas in the second column, a quadratic polynomial is used. The cuff-off point is 30 years of smoking (to the right of each graph are individuals who have smoked for at least 30 years, and to the left are smokers with less than 30 years of smoking).

## 4. Discussion

This paper investigated the effect of smoking duration on self-assessed health and smoking-related diseases among smokers using the South African National Income Dynamic Study (NIDS), which contains information on the current age and the age the respondent started smoking. This information enabled to clearly identify the treatment groups. While there has been a considerable amount of research on the health effect of smoking, there is limited evidence on the health effects of smoking duration [6,17,18,19]. This is particularly the case in developing countries where lack of data has hampered research in this area. These studies have major setbacks, including the focus on a limited number of smoking-related diseases and the exclusion of individual characteristics that are relevant in explaining variation in health. The study, therefore, considers comprehensively a number of smoking-related diseases and individual characteristics that are important in explaining variation in health status. Relatedly, the health profile of countries differs significantly, making it difficult to generalize findings from previous studies. Based on knowledge from literature review, no study has analysed this relationship in South Africa, and none of the existing studies has explored this relationship using an ATET or a RD design. The paper addresses this evidence gap by examining the hazardous effects of smoking duration on health.

Using ATET, RD, and OLS approaches, this paper documents that cigarette smoking is associated with a higher probability of reporting poor health and/or suffering from related diseases. The probability increases with the number of years of smoking, as the magnitude of the effect is larger at higher cut-off points. For example, the study found that relative to non-smokers, the probability of reporting poor health is slightly lower for individuals who have smoked for 20 to 29 years (3.6%) than 10 to 19 years (3.7%) but significantly higher for those who have smoked for over 30 years (4%). The parametric estimates suggest that smoking for over 20 years is associated with an increase in the composite index of smoking-related diseases and in the probability of reporting poor health. This is in line with the conclusion that the health damage from smoking usually emerges at least 20 to 30 years after exposure [16]. The study also found that smoking for less 10 years has no significant effect on the probability of reporting poor health across all waves but significant increases the index of the related disease profile between 0.31 and 0.47 units. While the significant effect of smoking duration on the related disease profile is consistent over time, the magnitude of the effects differs across the different survey waves used. A number of studies have also confirmed that number of cigarettes per day and years of smoking were each associated with Parkinson’s disease (PD) risk [41,42]. Chen et al. [17] showed that duration of smoking was more important than smoking intensity in modulating PD risk, and among past smokers, the lowest risk of PD was observed for participants who smoked the longest. It is also well-established that cigarette smoking is a single risk factor for chronic obstructive pulmonary disease (COPD) [43]. Pezzuto et al. [44] showed that extreme CYP2A6 phenotypes affect the rate of COPD occurrence among smokers. A critical evaluation of the dose–response relationship suggests that the odds ratio for lung cancer was greater with pack-years or duration than cigarettes per day [45]. Thus, obstructive lung disease also appears to be affected to a greater extent by smoking duration than intensity.

The paper also provides new estimates of the relationship between cigarette smoking and related diseases for different socioeconomic groups, which complements the existing literature. Evidence from the study suggests that smokers are significantly more likely to report poor health and/or diagnosed related diseases compared to non-smokers. The results suggest that the composite index of poor health ranges between 0.10 and 0.16 units higher for smokers, while the probability of reporting poor health ranges between 2 and 4 percentage points. The ATET by gender indicates that the index of poor health is between 0.09 to 0.15 units higher among male smokers and 0.13 to 0.17 for female smokers relative to their non-smoking counterparts. The study further estimated the impact of smoking on related diseases by race and level of education. The estimated indices of poor health are more highly significant among Black smokers than they are with coloured smokers and are insignificant for White smokers. The magnitude of the effect by education is inconsistent across the different waves. The estimates for those with primary education are slightly greater in magnitude in Wave 1 and Wave 2, while the estimates of those with tertiary education are greater in Wave 3 and Wave 4.

## 5. Limitations

Although this paper has documented the relationship between smoking and health, it could not include all smoking-related health outcomes due to the limitations of the data. In addition, it would have been interesting to include the pack-year index and information regarding second-hand smoking exposure, but the data do not contain information on the packs of cigarettes smoked. For further research to address this limitation, there is need for detailed survey data to include all smoking-related health outcomes.

## 6. Conclusions

Through this study, the impact of smoking and smoking duration on smoking-related diseases and self-assessed health was identified. Using both parametric and non-parametric methods, this paper documents that cigarette smoking is associated with a higher probability of reporting poor health and/or suffering from related diseases. The probability increases with the number of years of smoking, as the magnitude of the effect is larger at higher cut-off points. Therefore, if tobacco control policies can achieve the intended results of reducing cigarette smoking, the goal of preventing premature mortality from smoking-related diseases can be actualized, thereby creating public health benefits.

## Figures and Tables

**Table 1:** Descriptive statistics of key variables in Wave 4.

	All Individuals			Smokers Only		
Variable	Obs.	Mean	Std. Dev.	Obs.	Mean	Std. Dev.
Individual is a current smoker	22,727	0.203	0.403			
Have years of smoking for smokers				4267	17.34	0.332
Diagnosed of tuberculosis	21,937	0.035	0.184	4038	0.056	0.230
Diagnosed of high blood pressure	20,358	0.106	0.308	3897	0.098	0.297
Diagnosed of diabetes	22,034	0.036	0.187	4176	0.027	0.162
Diagnosed of stroke	22,575	0.009	0.096	4240	0.010	0.099
Diagnosed of a heart disease	22,356	0.018	0.134	4179	0.020	0.141
Diagnosed of cancer	22,648	0.013	0.114	4250	0.016	0.124
Have persistent cough	22,737	0.127	0.333	4265	0.168	0.374
Experienced depression	22,742	0.447	0.497	4263	0.505	0.500
Experienced chest pain	22,735	0.093	0.291	4264	0.128	0.334
Self-reported poor health	22,744	0.108	0.311	4262	0.126	0.332
Health index	19,440	0.026	1.247	3642	0.175	1.335

**Table 2:** Health effects of smoking duration.

	Wave 1		Wave 2		Wave 3		Wave 4	
Variables	SAH	Index	SAH	Index	SAH	Index	SAH	Index
≤10 years	0.025	0.101 **	0.020	0.160 ***	0.012	0.206 ***	0.012	0.128 ***
	(0.015)	(0.041)	(0.015)	(0.046)	(0.013)	(0.039)	(0.011)	(0.033)
10–19 years	0.023	0.091 **	0.012	0.141 ***	0.029 **	0.094 **	0.037 ***	0.150 ***
	(0.015)	(0.046)	(0.014)	(0.052)	(0.013)	(0.044)	(0.011)	(0.040)
20–29 years	0.026 *	0.250 ***	0.060 ***	0.378 ***	0.053 ***	0.155 ***	0.036 ***	0.116 **
	(0.014)	(0.050)	(0.015)	(0.057)	(0.013)	(0.050)	(0.011)	(0.049)
30+ years	0.065 ***	0.365 ***	0.028 **	0.474 ***	0.059 ***	0.386 ***	0.040 ***	0.313 ***
	(0.013)	(0.045)	(0.011)	(0.052)	(0.011)	(0.045)	(0.009)	(0.048)
Constant		−0.979 ***		−1.146 ***		−0.732 ***		−0.482 ***
		(0.099)		(0.096)		(0.091)		(0.075)
Observations	14,912	14,847	16,321	16,048	18,322	18,188	22,241	19,037
R-squared		0.106		0.129		0.139		0.100

Note: The base category for smoking duration is never smoked cigarettes. The estimation control is for some individual characteristics, including the quadratic of age, education, race, marital status, gender, and income. Standard errors in parentheses; *** *p* < 0.01, ** *p* < 0.05, * *p* < 0.10.

**Table 3:** The effect of smoking on health and by gender.

	Wave 1		Wave 2		Wave 3		Wave 4	
	Index	SAH	Index	SAH	Index	SAH	Index	SAH
ATET	0.142 ***	0.035 ***	0.139 ***	0.023 ***	0.164 ***	0.042 ***	0.103 ***	0.024 ***
	(0.029)	(0.009)	(0.032)	(0.008)	(0.028)	(0.007)	(0.026)	(0.006)
Observations	15,241	15,307	16,432	16,717	18,516	18,651	19,367	22,657
	Wave 1		Wave 2		Wave 3		Wave 4	
	Female	Male	Female	Male	Female	Male	Female	Male
ATET	0.133 ***	0.148 ***	0.132 **	0.150 ***	0.288 ***	0.135 ***	0.168 ***	0.093 ***
	(0.049)	(0.037)	(0.067)	(0.039)	(0.057)	(0.033)	(0.058)	(0.030)
Observations	9131	6111	9626	6806	11,031	7486	10,901	8467

Note: The results control for some individual and household characteristics, including the quadratic of age, educational, race, marital status, gender, and household per capita income. Standard errors in parentheses; *** *p* < 0.01, ** *p* < 0.05.

**Table 4:** The effect of smoking on health by race and by education.

		Wave 1			Wave 2			Wave 3			Wave 4	
	Black	Coloured	White	Black	Coloured	White	Black	Coloured	White	Black	Coloured	White
ATET	0.16 ***	0.13 **	0.14 *	0.19 ***	0.00	0.29 *	0.17 ***	0.09	0.31 **	0.10 ***	0.10 *	0.14
	(0.04)	(0.06)	(0.07)	(0.03)	(0.07)	(0.16)	(0.03)	(0.06)	(0.13)	(0.03)	(0.06)	(0.12)
Observations	12,001	2143	1097	13,718	2091	624	15,245	2555	716	16,345	2441	582
		Wave 1			Wave 2			Wave 3			Wave 4	
	Primary	Secondary	Tertiary	Primary	Secondary	Tertiary	Primary	Secondary	Tertiary	Primary	Secondary	Tertiary
ATET	0.24 ***	0.05	0.20 ***	0.16 **	0.12 ***	0.02	0.20 ***	0.11 ***	0.23 ***	0.15 **	0.08 ***	0.18 ***
	(0.05)	(0.03)	(0.08)	(0.06)	(0.04)	(0.10)	(0.06)	(0.03)	(0.08)	(0.07)	(0.03)	(0.07)
Observations	5811	8355	1076	5835	9492	1105	5941	10,862	1713	4526	12,585	2256

Note: The results control for some individual and household characteristics including the quadratic of age, marital status, gender, and household per capita income. Standard errors in parentheses; *** *p* < 0.01, ** *p* < 0.05, * *p* < 0.10.

## Data Availability

Not applicable.
